# Predictive hybrid scan-to-BIM method improves heritage building documentation completeness and accuracy

**DOI:** 10.1038/s41598-026-38200-8

**Published:** 2026-02-06

**Authors:** Rnin Salah, Nóra Géczy, Kitti Ajtayné Károlyfi

**Affiliations:** 1 Department of Structural and Geotechnical Engineering, Széchenyi István University, Győr, 9026 Hungary; 2 Department of Architectural Design, Széchenyi István University, Győr, 9026 Hungary

**Keywords:** Scan-to-BIM, Laser scanning, Photogrammetry, Predictive feasibility, Building documentation, Point cloud analysis, Hybrid integration, Engineering, Mathematics and computing

## Abstract

Incomplete survey data often undermines the reliability of Building Information Models (BIM), particularly for structures with restricted access and complex geometries. This study demonstrates a hybrid Scan-to-BIM workflow that integrates terrestrial laser scanning (TLS) and unmanned aerial vehicle (UAV) photogrammetry, supported by a predictive feasibility concept, to improve documentation accuracy and completeness. A two-phase strategy was validated on a chapel case study. Phase 1, combining TLS and ground-based photogrammetry, achieved only 54% coverage due to severe occlusions and limited scanner placement. These results led to the formulation of a Predictive Scan Feasibility Estimation Model (PSFEM), designed to generalize site-specific parameters such as scanner range, clearance angle, and building height into a decision-support tool for future surveys. Guided by the recognition of Phase 1 limitations, Phase 2 incorporated UAV photogrammetry and supplemental TLS, increasing coverage to 96%. Comparative analyses confirmed consistency in accuracy and improved geometric completeness. While the PSFEM was developed retrospectively based on the limitations identified in Phase 1, its analytical validation demonstrates the potential value of predictive planning for reducing redundant site visits and enhancing BIM reliability. The proposed framework provides a transferable basis for applying predictive hybrid workflows in both heritage and complex building documentation. This workflow offers a practical and scalable method for Scan-to-BIM documentation, applicable to heritage as well as other complex buildings, enabling high accuracy and completeness while effectively managing time and resources.

## Introduction

The rapid evolution of Historic Building Information Modeling (HBIM) has transformed how heritage professionals document, analyze, and manage culturally significant structures. By converting field-acquired geometric and visual data into parametric, queryable digital twins, HBIM supports condition assessment, structural analysis, preventive conservation, and long-term facility management, even extending to historic transportation infrastructure^1–4^. Recent reviews have also emphasized the growing integration of HBIM with other information systems such as GIS, photogrammetry, and digital twin environments to support comprehensive heritage documentation and decision-making workflows^5^. However, the overall reliability of HBIM deliverables is fundamentally governed by two intertwined attributes: accuracy and coverage. Even minor gaps in roof or façade geometry can propagate dimensional errors, undermine clash detection, and misinform conservation decisions^6,7^. Furthermore, Gerke et al.^8^ emphasized the importance of comprehensive, high-resolution documentation and demonstrated how incomplete point cloud coverage directly affects surface geometry accuracy in cultural heritage surveys. As a result, a growing body of research has focused on improving survey techniques, integrating data sources, and quantifying uncertainty to ensure HBIM completeness^9,10^.

Among reality-capture technologies, terrestrial laser scanning (TLS) and close-range photogrammetry remain foundational tools for heritage documentation. Aicardi et al.^11^ extensively reviewed photogrammetric computer-vision approaches, showing how dense imagery can supplement TLS data to improve both geometric completeness and textural fidelity, especially in decorative or occluded zones often missed by scanners. Crucially, ensuring the effective transformation of this acquired data into a usable HBIM, especially through structured Scan-to-BIM methodologies and adherence to heritage conservation standards, is also a critical area of ongoing framework development^12,13^. TLS delivers sub-centimeter metric precision and consistent point density, making it indispensable for structural monitoring and deformation studies^14^. Photogrammetry offers true-color texture, flexible acquisition geometries, and lower hardware costs, making it particularly useful in visually complex or budget-constrained projects^15^. Numerous case studies have demonstrated the value of TLS–photogrammetry integration, where the strengths of one method offset the limitations of the other^16–20^.

Despite their combined strengths, these technologies often face critical site-specific constraints. Narrow urban passages, dense vegetation, and adjacent buildings can block scanner lines of sight; reflective materials degrade photogrammetric alignment; and safety regulations frequently limit elevated tripod or cherry-picker usage^2,21^. Consequently, essential elements like high roofs, hidden cornices, or rear apses often remain undocumented, forcing costly return visits or resulting in incomplete HBIM models.

In recent years, drone-based (UAV) photogrammetry has emerged as a powerful complement to traditional methods, capturing nadir and oblique imagery of hard-to-reach areas such as spires, towers, and steep roofs^22,23^. Hybrid TLS–UAV workflows have achieved near-total coverage and sub-decimeter accuracy for a range of heritage typologies including churches, castles, and archaeological sites^24,25^. However, drone flights are typically scheduled reactively, only after on-site inspections or initial alignments reveal gaps. This “scan-first-fix-later” approach introduces redundant mobilization, increases costs, and contradicts conservation best practices that prioritize minimal site disturbance.

Some research has attempted to improve pre-survey planning through heuristic checklists or field experience-based protocols^26,27^. Yet, these approaches lack generalizability, remain qualitative, and are rarely validated against quantitative coverage metrics. Concurrently, predictive modelling and simulation strategies have begun emerging in related fields such as robotics and mobile mapping, employing voxel-based occlusion analysis, sensor models, and digital twins for proactive capture planning^28–30^. Some frameworks have even explored BIM-supported autonomous LiDAR-carrying UAVs, integrating scan coverage planning with motion planning for efficient trajectories and high coverage^27^. Still, their translation into the HBIM domain remains limited.

One promising direction lies in integrating geometric modeling and survey simulation tools into HBIM planning frameworks. For instance, Mahmood et al.^31^ demonstrated how geometric constraints and scanner parameters can be modeled to predict data coverage and quality, forming the foundation for proactive HBIM survey planning. By developing mathematical models that relate scanner parameters to target surface characteristics, these tools can guide scanner placement to minimize geometric distortions and optimize scan results^32^. Likewise, Huang et al.^33^ introduced effective scanning range estimation methods that predict TLS coverage limitations in advance, based on building height and scanner specifications, thus enabling survey teams to anticipate occlusions and plan accordingly. Virtual planning environments also offer a means to simulate scan coverage prior to field deployment, as demonstrated by Luhmann et al.^34^, who developed a VR-based scanning simulator to optimize scanner placement and reduce trial-and-error during scan execution.

Despite these advancements, there remains a gap in the HBIM literature: no standardized, quantitative model has been widely adopted to assess scan feasibility prior to data collection. Such a model could empower heritage teams, particularly those with limited budgets or field access, to evaluate in advance whether drone-based acquisition is necessary, potentially consolidating ground and aerial data collection into a single optimized visit.

The 19th-century chapel of Sopronhorpács, Hungary, exemplifies these challenges. Surrounded by dense vegetation, narrow access paths, and adjacent structures, the site restricted scanner placement and vertical visibility. A preliminary Phase 1 survey using TLS and ground-based photogrammetry achieved only 53.6% coverage, leaving roof ridges, tower caps, and drainage elements undocumented. These limitations highlighted the need for a more structured and analytically informed approach to assessing scan feasibility.

The rest of this paper is organized as follows: Section “Research aim” outlines the research aim of the study. Section “Materials and Methods” describes the phased hybrid acquisition methodology and details the theoretical foundation of the Predictive Scan Feasibility Estimation Model (PSFEM) and describing the data processing and evaluation metrics used across the two survey phases. Section “Results and comparative analysis” presents the case study results, focusing on accuracy, density, and complete comparisons between the two acquisition phases. Section “Discussion and Implications for HBIM Practice” discusses the practical implications of the findings, including limitations and future opportunities for AI-enhanced registration and occlusion handling. Section “Proposed Decision-Making Framework” proposes a decision-making framework for phased survey planning in heritage documentation. Section “Conclusion” concludes with key findings and recommendations for future development of predictive HBIM workflows.

## Research aim

This study aims to introduce and implement a predictive, phased hybrid data acquisition workflow for HBIM documentation in complex heritage sites. The proposed approach combines ground-based and aerial data acquisition within a structured two-phase process designed to improve spatial coverage while maintaining efficiency in time and resource use.

A key outcome of this research is the development of the PSFEM, which emerged directly from limitations observed during Phase 1 of the survey. Rather than guiding the initial data acquisition, PSFEM was formulated retrospectively, based on actual site conditions such as scanner placement constraints, building geometry, and field-of-view limitations. As such, the model is analytically validated through the case study and is intended as a transferable decision-support tool to support early-stage planning in future HBIM workflows.

The proposed workflow is demonstrated through a case study of the Sopronhorpács chapel, where the hybrid strategy increased geometric coverage from 53.6% to 96.3% and point density from 49 to 89 million points while maintaining geometric accuracy. Beyond validating the effectiveness of phased ground–aerial integration, the study contributes a structured and predictive decision-making framework that supports more efficient, informed, and resource-aware heritage documentation practices.

## Materials and methods

### Case study: historic Chapel church in Sopronhorpács

The selected case study is the Sopronhorpácsi Szent Péter és Pál-templom (St. Peter and Paul Church in Sopronhorpács), a historic chapel known for its significant age and various construction stages over time, requiring precise documentation. Its foundations were originally laid in the 1230s, and the church has undergone multiple rebuilds throughout centuries, yet these early structures still provide its core stability. According to current architectural understanding, it represents a unique “hybrid style,” faithfully reflecting the changes of successive historical periods. This makes it an architecturally interesting and historically rich monument.

**Fig. 1:**
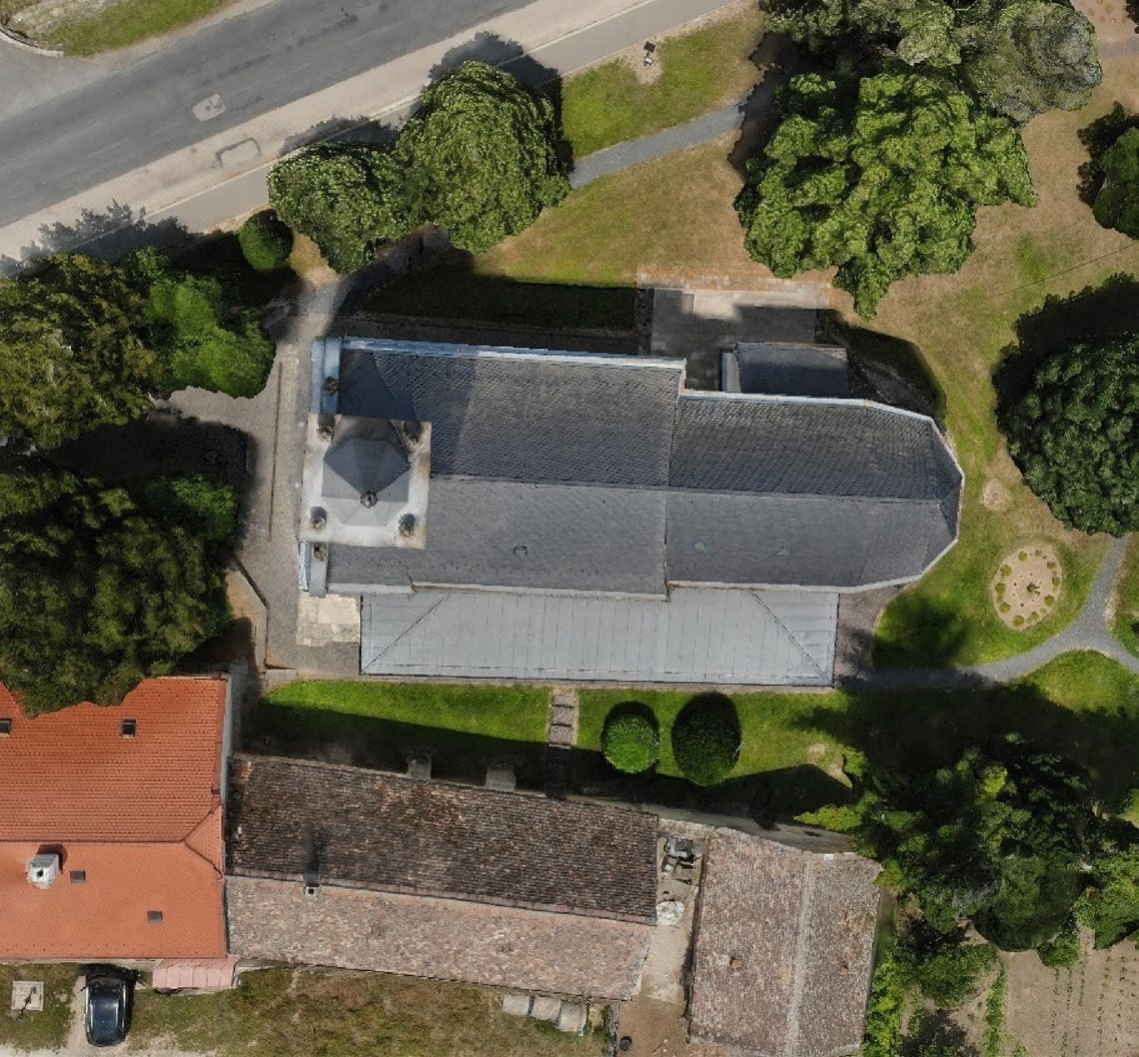
Site constraints at the chapel (narrow lanes, vegetation) limiting scanner placement. *Image credit: photograph by R. Salah, CC BY 4.0.*

The site itself presents notable challenges for documentation. It is surrounded by narrow pathways, dense vegetation, and adjacent structures, which severely limit viable scanner placement and camera angles during ground-based surveys (Fig. 1). These constraints make it an ideal case for testing a phased hybrid data acquisition approach to improve HBIM documentation, as a single ground-based survey could not achieve the desired completeness.

### Hybrid multiphase workflow overview

This study implements a two-phase hybrid data acquisition strategy to enhance the geometric accuracy and spatial completeness of 3D documentation for HBIM. By combining TLS, ground-based photogrammetry, and aerial-based photogrammetry, the workflow uses the complementary strengths of each technology to overcome the limitations imposed by complex site conditions.

**Fig. 2:**
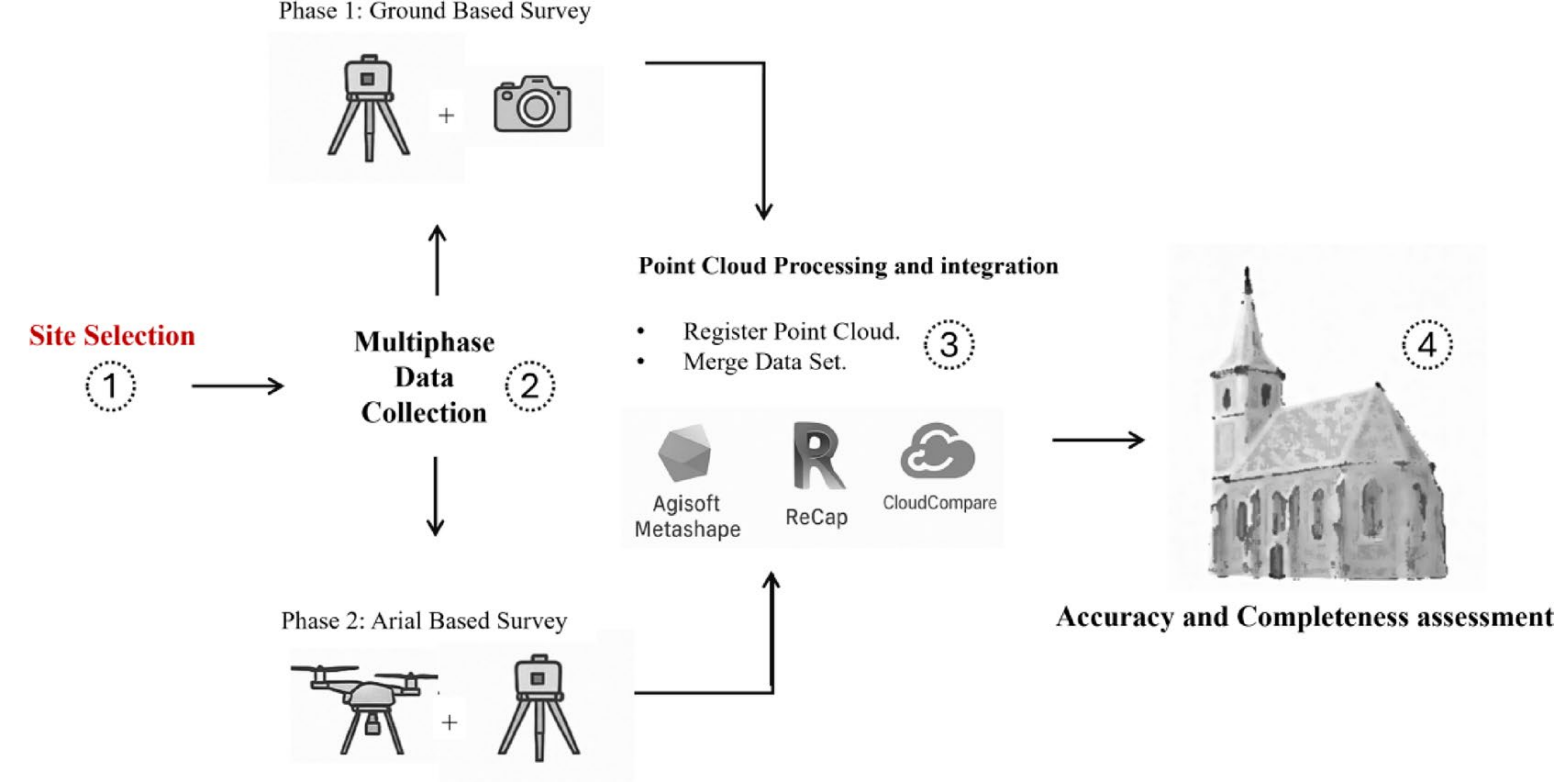
Hybrid multiphase workflow for HBIM documentation.

The overall workflow consists of four main components (Fig. 2):

1. Site selection and survey design.
2. Multiphase data collection.
3. Point cloud processing and integration.
4. Accuracy and completeness assessment.

Phase 1 involved TLS and ground-based photogrammetry, targeting the most accessible areas of the building. However, due to significant occlusions and constraints on scanner placement, the resulting documentation lacked sufficient coverage. This outcome highlighted the limitations of relying solely on ground-based methods in constrained heritage sites.

The analysis of Phase 1 results led to the development of a PSFEM. This model, formulated as a retrospective byproduct of this study, quantitatively relates scanner range, building height, and clearance angle to assess the likelihood of achieving full coverage using only ground-based techniques. It is important to emphasize that the PSFEM was not applied prospectively to plan the surveys in this project; instead, it was derived from the observed site constraints to serve as a transferable decision-support tool for future HBIM survey planning.

Following the coverage analysis, Phase 2 was executed to address the identified gaps. This phase incorporated aerial-based photogrammetry and supplemental TLS to capture previously inaccessible rooflines, tower caps, and elevated features. The integration of this second phase resulted in a significant improvement in completeness while preserving data accuracy and minimizing redundant effort.

### Multiphase data acquisition strategy

#### Phase 1: initial survey, ground-based data capture

The first phase of data collection was conducted using a Leica HDS-6200 terrestrial laser scanner and ground-based photogrammetry with two types of smartphone cameras: Samsung Galaxy A52s (735 images) and iPhone 14 Pro Max (456 images). TLS is widely recognized for its high metric accuracy in cultural heritage documentation^35^, while image-based modeling with smartphones offers flexibility and cost-efficiency in heritage environments^36^. This phase aimed at producing a complete, high-fidelity base dataset suitable for integration into HBIM workflows^12^. To identify the optimal photogrammetric dataset for integration with the TLS data, ten structured trials were conducted.

These trials systematically tested various image counts, capture strategies, and processing software configurations. Each trial was evaluated based on two criteria: model quality and geometric accuracy, both scored on a 1–5 scale through visual inspection. The results of these trials, presented in Table 1, guided the selection of the dataset that provided the best balance between accuracy and manageable data size for Phase 1 integration. The evaluation revealed that photogrammetric model quality did not exhibit a direct correlation with the number of input images. Trials with fewer but strategically captured photographs, such as Trial 9 using only 100 images, produced models of higher geometric integrity and alignment precision than those with significantly larger datasets, including Trial 8 with 834 images. This finding underscores the importance of informed image acquisition strategies, such as angle diversity and coverage efficiency, over sheer image volume, particularly in heritage environments where visual access is constrained.

**Table 1:** Evaluation summary of photogrammetry trials. *Image credit: photograph by R. Salah, CC BY 4.0.*

Trial	Software	Images	Device	Model quality	Accuracy	Main challenge	Model quality score	Accuracy score	Perspectives
1	Agisoft	456	iPhone	Medium	Medium–High	Missing roof side, upper tower, upper façade details	3	4	
2	Agisoft	438	iPhone	Low	Medium	Incomplete roof, missing upper tower & façade parts	1	3	
3	Agisoft	100	iPhone	Low-Medium	Medium–High	Incomplete roof & tower; missing upper façade details	2	4	
4	Agisoft	309	iPhone	Medium	Medium–High	Incomplete roof & tower; missing upper façade details	3	4	
5	Agisoft	145	iPhone	Medium	Low-Medium	Incomplete roof & tower; missing upper façade details	3	2	
6	Agisoft	200	iPhone	Medium	Medium–High	Incomplete roof & tower; missing upper façade details	3	4	
7	Agisoft	136	iPhone + Android	Medium	Medium	Incomplete roof & tower; missing upper façade details	3	3	
8	Agisoft	834	iPhone + Android	Medium	Medium	Incomplete roof & tower; missing façade details	3	3	
9	Agisoft	100	iPhone	High	High	Some roof & tower parts missing; incomplete upper façade	5	5	
10	ReCap	100	iPhone	High	High	Some roof & tower parts missing; incomplete upper façade	5	5	

To further support the qualitative assessment of geometric completeness, two representative perspectives from each model were selected to visually assess surface completeness, particularly in occlusion areas such as the roof, cornice, and tower caps.

Despite the relative success of Trials 9 and 10, none of the tested datasets achieved full reconstruction of upper architectural features. Coverage limitations remained particularly evident in the roof ridges, tower caps, and cornice areas. These gaps were not a result of image quantity or processing parameters but rather stemmed from constrained camera viewpoints caused by the site’s narrow access paths, adjacent structures, and surrounding vegetation.

**Fig. 3:**
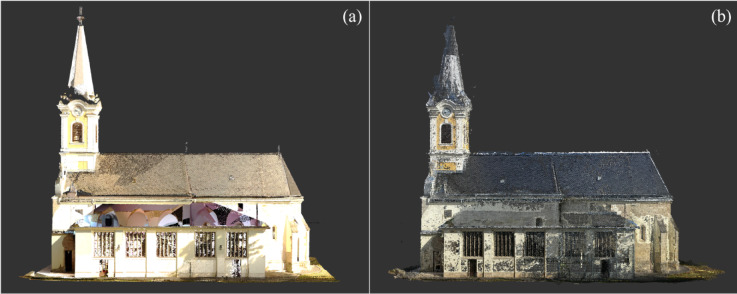
Ground-based Data Sets: (**a**) TLS1 Data Set (**b**) Phone Photogrammetry Data Set. *Image credit: photograph by R. Salah, CC BY 4.0.*

Although this phase yielded dense and high-resolution data for ground-level features, it became evident that the upper parts of the structure, especially the roof and tower areas, as mentioned before remained incomplete due to inherent occlusions and severely restricted camera and scanner angles imposed by the site specifications^31,33^, resulting in approximately 54% coverage of the building geometry in this phase (Fig. 3).

These observed limitations were then analyzed and led us to consider a custom survey feasibility estimation model.

#### Phase 2: complementary survey, aerial-based data capture

**Fig. 4:**
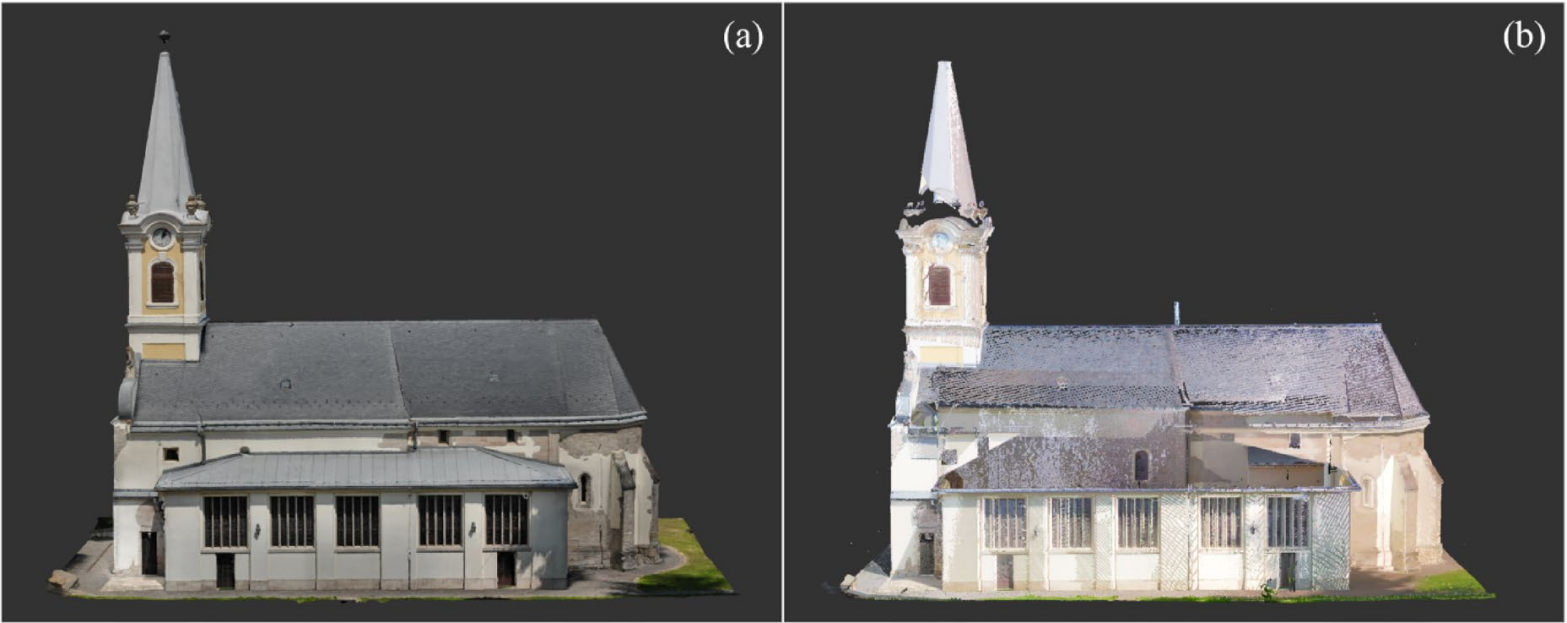
Aerial-based Data Sets: (**a**) Drone Photogrammetry Data Set (**b**) TLS 2 Data Set. *Image credit: photograph by R. Salah, CC BY 4.0.*

Following the findings and coverage analysis from Phase 1, Phase 2 was specifically planned to address the significant data gaps caused by the limitations of ground-based surveys, particularly in documenting upper structures^37^. A DJI Mavic 2 Enterprise Dual drone, equipped with a high-resolution camera, was deployed to capture 1549 oblique and nadir images, ensuring comprehensive coverage of sloped, shadowed, and otherwise inaccessible roof and upper façade areas. To further enhance completeness and registration accuracy in these challenging zones, supplemental TLS scans were conducted using a Leica BLK360 G2 scanner. This scanner was intentionally used to test whether a different TLS type could provide improved data quality and geometric accuracy, complementing the drone-based photogrammetry outputs^38,39^. This dual strategy in Phase 2, integrating aerial photogrammetry with additional TLS, successfully increased the documented geometry to approximately 96% coverage (Fig. 4), ensuring a sufficiently complete and high-fidelity dataset for HBIM modeling and further structural or conservation analysis.

### Data processing and integration workflow

All point cloud processing and integration were conducted in CloudCompare to ensure consistency, precise alignment, and traceable data cleaning across both phases. The workflow included importing TLS and photogrammetry datasets, applying noise filtering and subsampling, aligning using the ICP (Iterative Closest Point) algorithm, and merging to generate comprehensive, high-fidelity point clouds suitable for HBIM^40^.

Phase 1 focused on capturing detailed façade geometry by integrating TLS and photogrammetry data, while Phase 2 incorporated drone photogrammetry and supplemental TLS to document upper and previously occluded areas. Finally, both phases were integrated to produce a unified dataset that balances geometric accuracy with near-complete coverage under the stated constraints for HBIM documentation.

An overview of these steps is provided in Table 2.

**Table 2:** Summary of data processing and integration workflow.

Step	Details
Phase 1 processing	TLS Data Import and Pre-processing: The point cloud from the Leica HDS-6200 laser scanner was imported. XYZ directions were adjusted to align with the project’s coordinate system Photogrammetry Data Processing and Optimization: 10 trials were conducted in Agisoft Metashape and ReCap photo, with Trial 9 identified as optimal, using only 100 carefully selected iPhone images. This choice was made not based on the quantity of images but on the high accuracy and detailed wall geometry achieved while maintaining a manageable data size. To further enhance coverage and data quality, the best outputs from additional selected trials were also merged, resulting in a combined photogrammetry point cloud of 295,280,474 points Noise Filtering and Subsampling (Photogrammetry): A Radius Noise filter was applied to the photogrammetry point cloud, reducing it to 197,753,019 points. Subsequently, a Distance-based Subsampling Tool (with a distance of 0.001) was used to remove duplicated points, resulting in 31,439,929 points Alignment and Merging: Both the processed TLS point cloud (18,113,861 points) and the subsampled photogrammetry point cloud were imported and aligned. The ICP algorithm was used for precise registration. The final merged Phase 1 point cloud contained 49,553,790 points
Phase 2 processing	Drone Photogrammetry Processing: Images from the DJI Mavic 2 Enterprise Dual drone were processed using Agisoft Metashape, resulting in a 100% complete photogrammetry model. The initial point cloud, including the church and its environment, contained 78,482,919 points. This was then cleaned to isolate only the church, yielding 14,975,218 points Supplemental TLS Data Processing: The point cloud from the Leica BLK360 (G2) laser scanner was imported, and its XYZ directions were adjusted. This initial scan contained 106,986,856 points. A Radius Noise filter was applied, reducing the count to 76,298,656 points. Further subsampling (Distance-based, 0.001) was performed to remove duplicates, resulting in 74,098,714 points Alignment and Merging (Phase 2): The cleaned drone photogrammetry point cloud and the processed supplemental TLS point cloud were imported and aligned using ICP. The final merged Phase 1 point cloud contained 89,073,932 points
Final hybrid integration	The final comprehensive HBIM model was created by integrating the merged point clouds from both Phase 1 and Phase 2. This involved: Initial Alignment: The Phase 1 merged cloud and the Phase 2 merged cloud were aligned using manual registration followed by ICP refinement. For the Phase 1 vs Phase 2 C2C comparison, the Phase 2 merged point cloud was chosen as the reference Statistical Filtering: Overlapping points between the two-phase datasets were filtered to reduce noise and ensure seamless transitions Model Merging: The combined dataset formed the final hybrid model, leveraging the accuracy of ground-based data with the completeness offered by aerial and supplemental scans, particularly in occluded and elevated regions. The final merged dataset contained 92,523,738 points

### Predict then capture: development of PSFEM model

The first phase of data acquisition employed TLS and ground-based photogrammetry to document the accessible areas of the chapel. However, subsequent data processing and visual inspection revealed substantial blind spots, particularly across the upper elements. These omissions were primarily caused by site constraints that restricted scanner placement and limited the vertical field of view due to adjacent buildings and narrow pathways. Despite attempts to optimize scanning positions, overall surface coverage did not exceed 53.6%, falling short of the completeness required for reliable HBIM documentation.

These limitations highlighted the inefficiency of the conventional “scan-first-then-adjust” approach, prompting a re-evaluation of the survey workflow. Rather than proceeding with additional field visits based on observational judgment alone, the team adopted a revised strategy: Predict Then Capture. This concept emerged as a direct response to the Phase 1 results and proposed a more systematic approach to determining whether ground-based documentation would be sufficient, or if aerial methods would be required to achieve full coverage.

To support this strategy, a PSFEM was developed. Notably, the model was formulated as a product of Phase 1 insights, it did not inform the original survey plan but instead arose from the need to quantify coverage feasibility in similar contexts moving forward. By linking building geometry with scanner positioning constraints, the model provides an objective basis for evaluating the potential visibility of elevated architectural elements from ground level.

**Fig.5:**
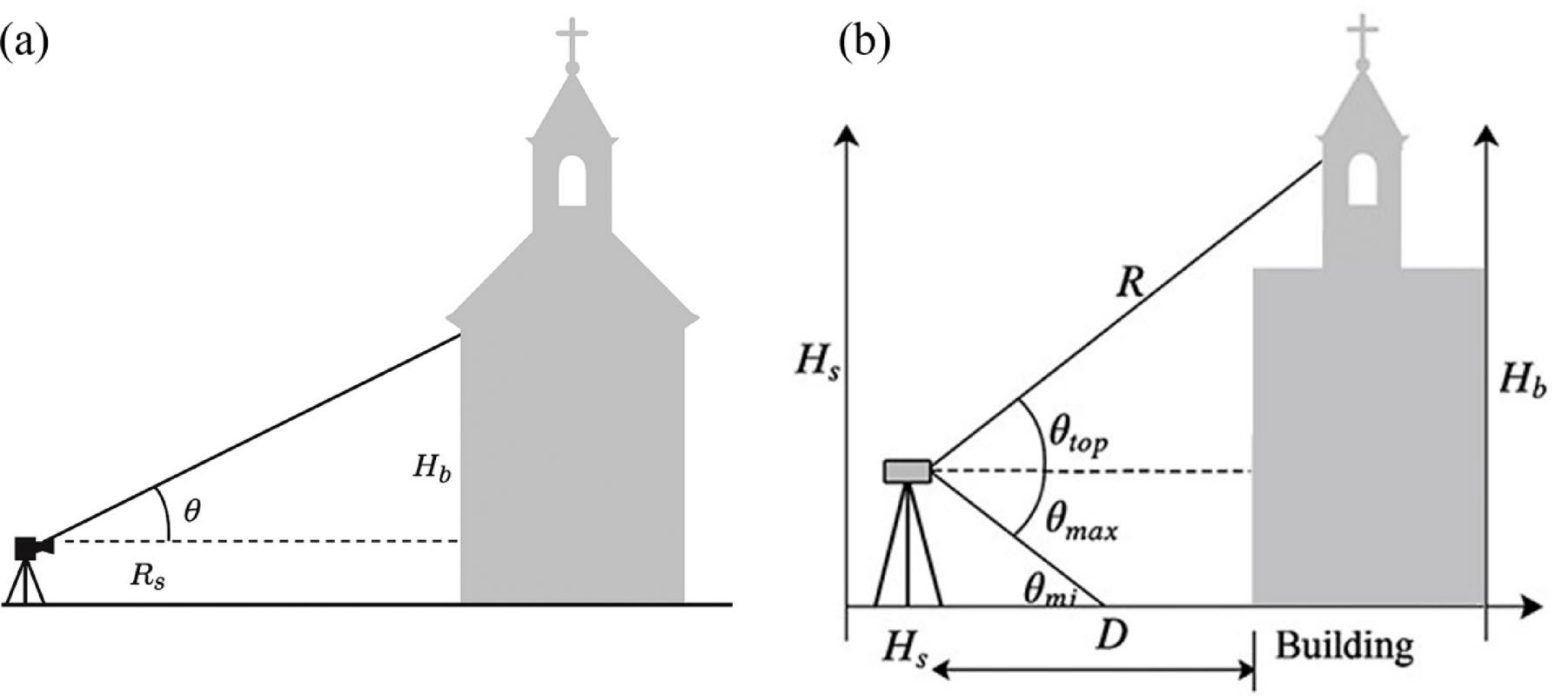
Calculation approaches for the PSFEM model: (**a**) Scan-Fit Index (SFI) and (**b**) Field of View (FOV).

PSFEM Formulation, two calculation approaches proposed, differing in complexity and required input parameters (Fig. 5):

1. Simplified SFI, analysis and parameters.

A compact, dimensionless formulation evaluates whether a scanner’s effective range and angular clearance are sufficient to capture the full vertical extent of the target structure, the model uses:

1 $$SFI = \frac{{R_{s} x sin\left( \theta \right)}}{{H_{b} }}$$

where $SFI$ represents the Scan-Fit Index, ${R}_{s}$ represents the effective scanner range (in meters), *θ* is the available clearance angle (in degrees), and ${H}_{b}$ refers to the building height (in meters).

Interpretation:

*SFI* ≥ 1 Sufficient visibility for scanning, the scanner can “see”; *SFI* < 1 occlusion likely, aerial survey recommended.

2. Full FOV Feasibility, analysis and parameters.

This formulation incorporates more detailed scanner parameters and site-specific geometry, Using measured site-specific parameters,${H}_{s}$ refers to the scanner height from the ground (in meters), D represents the horizontal distance from the scanner to the building, ${H}_{b}$ is the building height, ${\theta }_{min}$ and ${\theta }_{max}$ indicate the minimum and maximum vertical FOV of the scanner (in degrees), respectively. Finally, ${R}_{min}$ and ${R}_{max}$ specify the scanner’s minimum and maximum operational ranges (in meters), which determine the limits within which accurate data can be captured. Using these parameters, the following conditions are checked:

2 $$\theta_{top} = tan^{ - 1} \left( {\frac{{H_{b} - H_{s} }}{D}} \right)$$

3 $$\theta_{bottom} = tan^{ - 1} \left( {\frac{{0 - H_{s} }}{D}} \right)$$

4 $$R = \sqrt {(H_{b} - H_{s} )^{2} + D^{2} }$$

The building is considered fully scannable from the ground position if:

$$\theta_{top} \le \theta_{max} , \theta_{bottom} \ge \theta_{min} , R_{min} \le R \le R_{max}$$

Applying this model with parameters reflecting the actual site conditions (e.g., a small D value due to the narrow surrounding pathways and the relatively tall structure), reliably resulted in:

is_building_fully_scannable: False.

In quantitative terms, Phase 1 findings and established a solid geometric justification for proceeding to Phase 2. The integration of drone-based photogrammetry and supplemental TLS in the second phase was no longer based on observation alone, but grounded in a repeatable, analytical assessment of scan feasibility.

Although the model was not part of the original planning process, its development marks a critical shift toward data-informed heritage documentation. The PSFEM offers a practical, transferable tool that enables HBIM practitioners to anticipate documentation gaps in advance, optimize equipment selection, and streamline fieldwork into a single coordinated campaign when possible. This outcome-driven approach aligns with emerging best practices that prioritize predictive analytics and resource-efficient survey planning.

### Evaluation of metrics and analysis

The effectiveness of the developed multiphase hybrid workflow was evaluated using quantitative and qualitative metrics, ensuring the dataset’s reliability for HBIM modeling^41–43^. The evaluation focused on four key areas: accuracy, point cloud density, completeness (coverage), and surface roughness. These metrics were used to systematically compare Phase 1 and Phase 2 outputs and validate the benefits of hybrid integration.

- Accuracy was assessed using cloud-to-cloud (C2C) deviation analysis, calculating the mean, standard deviation, and root mean square (RMS) error between datasets. Three comparisons were made:
  1. Between TLS and ground-based photogrammetry (Phase 1),
  2. Between drone-based photogrammetry and TLS (Phase 2), and
  3. Between the merged datasets of Phase 1 and Phase 2 (with Phase 2 as the reference).
- Point cloud density was evaluated by calculating the total number of points in the merged point clouds after cleaning and filtering during each phase, providing insight into the richness and detail of the data.
- Completeness (coverage) was quantified as the percentage of the structure documented in each phase relative to the final merged Phase 1 + Phase 2 dataset. This metric represents a relative measure of spatial completeness rather than an absolute geometric reference. While it provides a consistent basis for comparing the contribution of each acquisition phase, it may be influenced by variations in point density and residual occlusions. Nevertheless, in the absence of an external ground-truth model, the final merged dataset represents the most comprehensive available representation of the site and serves as a practical and transparent baseline for evaluating improvements achieved through the hybrid workflow.
- Surface roughness analysis was used to assess local variations in the point clouds, helping to identify noise levels and the degree of detail preservation across surfaces.

Together, these metrics provided a comprehensive assessment of the hybrid workflow’s performance and its contribution to enhancing HBIM documentation quality.

## Results and comparative analysis

Using the previous evaluation metrics and analysis, this section presents the results of the phased hybrid workflow, contrasting Phase 1 (ground-based survey) with Phase 2 (drone-enhanced survey), emphasizing improvements in accuracy, density, completeness, and surface quality.

### Accuracy assessment

**Fig. 6:**
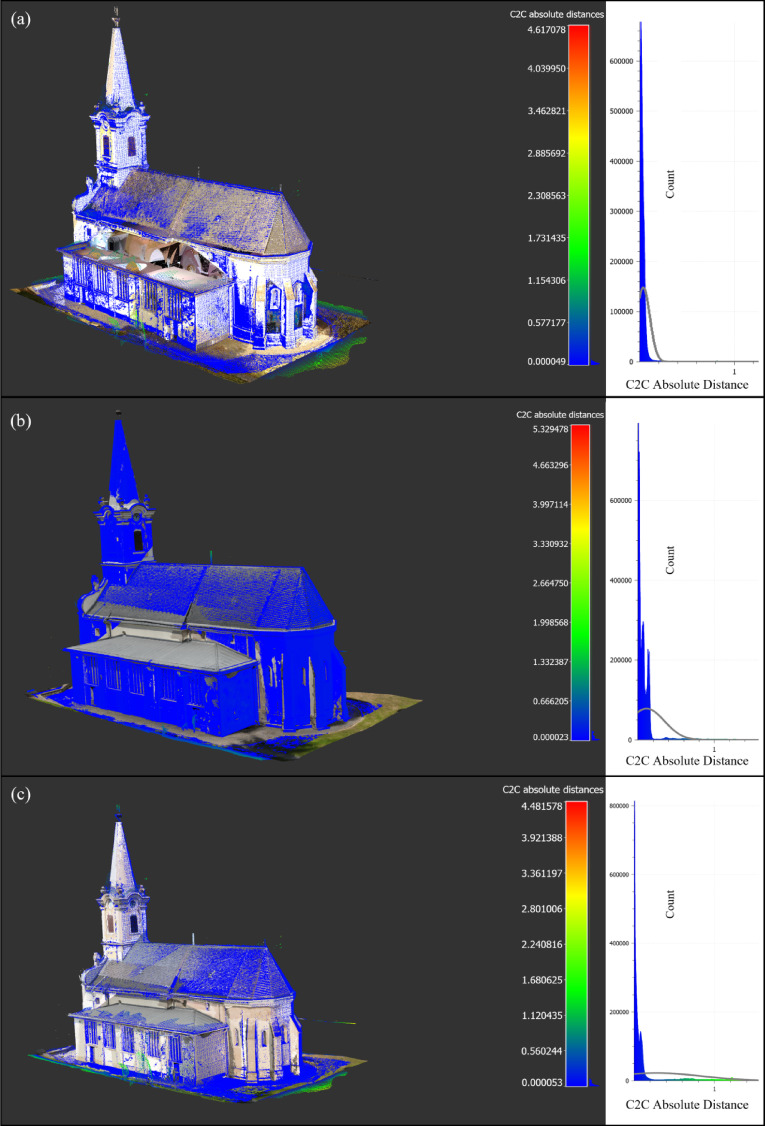
C2C Deviation Analysis and Distributions across Survey Phases (**a**) Phase 1 (TlS 1 as reference), (**b**) Phase 2 (TlS 1 as reference), and (**c**) Phase 1 vs Phase 2 (Phase 2 as reference).

Accuracy was evaluated using C2C deviation analysis in CloudCompare^44^, providing a quantitative measure of geometric alignment and highlighting areas of improvement across phases and, following three comparison strategies (Fig. 6):

- TLS 1 (as reference) and ground-based photogrammetry (Phase 1):

The mean distance between the ground-based photogrammetric model and the TLS reference was 38.3 mm, with a standard deviation of 69.6 mm and an RMS of 79.4 mm. This reflects the reasonably high alignment quality achievable from ground-based photogrammetry within accessible areas, despite limitations in documenting elevated structures.

- Drone photogrammetry (as reference) and TLS 2 (Phase 2):

The mean distance between the drone-based photogrammetric model and the Phase 2 TLS scan was 114.1 mm, with a standard deviation of 232.7 mm and an RMS of 259.2 mm. The higher deviation in Phase 2 is attributed to the complexity of the newly captured areas, including slopy roofs and intricate tower features, which are inherently challenging for precise alignment but crucial for completeness.

- Phase 1 and Phase 2 merged datasets (Phase 2 as reference):

The comparison revealed a mean distance of 280.7 mm, with a standard deviation of 554.6 mm and an RMS of 621.5 mm. This substantial deviation underscores the significant new geometric information captured during Phase 2, highlighting areas that were previously undocumented or poorly represented in Phase 1, thereby evidencing the improvement in dataset completeness.

Importantly, the increased C2C deviation values observed in Phase 2 do not indicate reduced measurement accuracy. Rather, they reflect the inclusion of newly captured architectural geometry, such as roof surfaces, tower caps, and upper façade regions, that were absent from the Phase 1 dataset. In phased acquisition workflows, such deviations are an expected outcome when spatial completeness increases. They should therefore be interpreted as indicators of geometric expansion rather than as evidence of reduced data quality.

### Point density and surface quality

The hybrid workflow led to a substantial increase in point density and improvement in surface quality across the entire model. The final merged Phase 1 point cloud contained 49,553,790 points. In contrast, the final merged Phase 2 point cloud, incorporating filtered high-angle drone data and supplemental TLS, contained 89,073,932 points. This represents a significant increase in data volume and detail.

Post-processing and statistical filtering, applied during the merging of both intra-phase and inter-phase datasets, led to a reduction in surface noise, particularly on large planar surfaces such as the roof and tower facades. The merged dataset preserved the geometric detail provided by TLS while enhancing resolution and texture through photogrammetry, confirming the effectiveness of the combined drone and TLS approach in enhancing data quality.

### Completeness and coverage enhancement

Model completeness was assessed through coverage analysis, using the number of valid points in each dataset relative to the total points in the fully merged Phase 1 + 2 dataset (92,523,738 points) as the reference for full coverage. This quantification was performed to evaluate spatial completeness before and after drone integration:

5 $$Coverage~\left( \% \right) = \left( {\frac{{Valid\;Points\;in\;Covered\;Areas}}{{Total\;Points\;for\;Full\;Coverage}}} \right) \times 100$$

Using CloudCompare’s density scalar fields and occlusion analysis, Phase 1 achieved 53.6% coverage, with significant gaps in roof structures and upper façades due to occlusions and site constraints that limited scanner placement and camera angles. Following the integration of drone-based photogrammetry and supplemental TLS in Phase 2, coverage increased to 96.3%, effectively reducing occlusions and enabling near-complete geometric documentation of the chapel. Previously undocumented features, including roof structures, tower caps, and drainage elements, were successfully reconstructed, resulting in a substantially more comprehensive dataset for HBIM documentation and modeling.

It should be noted that this coverage metric represents a relative measure based on the proportion of valid points within the final merged dataset, rather than an absolute measure of geometric completeness. As such, it is influenced by factors such as point density distribution, filtering strategies, and the spatial characteristics of the acquired data. Nevertheless, this approach provides a consistent and practical means of comparing documentation completeness between survey phases. In this context, the substantial increase in coverage reflects a meaningful improvement in spatial completeness rather than a definitive measure of total geometric capture.

### Visual assessment

**Fig. 7:**
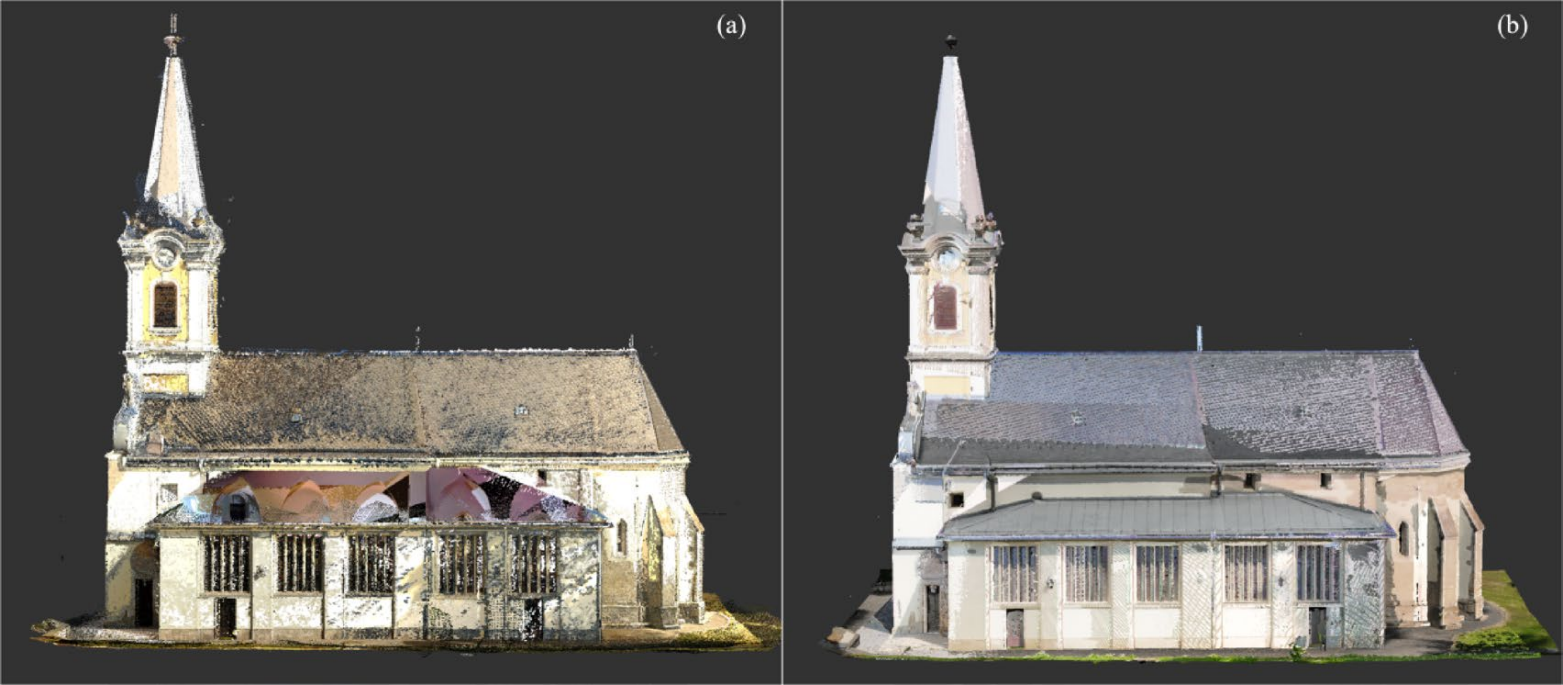
Side-by-side visualization of the (**a**) Phase 1 and (**b**) Phase 2 merged point clouds.

Visual comparisons of the point clouds further confirmed the quantitative results as shown in Fig. 7:

- Phase 1 dataset: Showed incomplete documentation of roofs and upper façades, with visible gaps in high-angle areas.
- Phase 2 dataset: Demonstrated complete and consistent documentation of complex architectural elements, including roof edges, tower caps, and drainage structures, with clear, high-density coverage.

### Summary of improvements

The multiphase hybrid workflow guided by predictive feasibility analysis resulted in:

- A 42% increase in coverage (from 54 to 96%).
- A 79.8% increase in point density (from 49.5 million to 89.1 million points).
- The capture of critical geometric details in previously inaccessible areas.
- Improved surface smoothness and reduced noise, enhancing the reliability of the HBIM model for heritage conservation applications.

The results confirm that the predictive, phased hybrid acquisition strategy significantly enhances HBIM data quality, addressing initial gaps due to site limitations and capturing critical geometric information. The integration of drone-based photogrammetry and supplemental TLS, informed by feasibility analysis, demonstrates a scalable and effective approach for heritage documentation in complex environments, providing high-fidelity data suitable for conservation, analysis, and long-term heritage management.

## Discussion and implications for HBIM practice

This study shows that using a predictive, step-by-step hybrid data collection strategy, based on early understanding of site limitations, greatly improves the accuracy, completeness, and reliability of HBIM documentation for heritage buildings.

The insights gained from this study, particularly the identified need for a second stage of data collection, strongly support the development and future application of models like PSFEM. This model was developed as a result from Phase 1. Its formulation was based on measured visibility gaps and scanner limitations observed on site. The model demonstrates how future surveys could be planned more efficiently, using measurable parameters to guide whether drone photogrammetry or additional TLS will be needed in advance.

Drone-based photography was very valuable for getting data from high-up and hard-to-reach parts. These areas are often very hard or even impossible to record well with only ground-based methods because things block the view and limit where you can stand^31,33^. Adding drone pictures greatly increased how much of the roof and upper parts of the building were covered, from about 53.6% to a nearly complete 96.3%^45^. This led to a highly complete dataset suitable for HBIM applications and aligns with previous studies reporting significant improvements in completeness when combining TLS and UAV data^46,47,48^.

While the mean C2C deviation in Phase 2 (114.1 mm) was higher than in Phase 1 (38.3 mm), this reflects the challenges of capturing newly documented and geometrically complex elements rather than a reduction in data quality. The larger deviation between the Phase 1 and Phase 2 datasets (280.7 mm) further confirms that Phase 2 introduced new geometric information rather than duplicating existing data. Importantly, these higher deviation values do not indicate reduced measurement accuracy but instead reflect the inclusion of previously undocumented architectural elements. As the workflow progressed from ground-based acquisition to a hybrid approach, additional areas became part of the dataset. In phased acquisition workflows, such behavior is expected, as spatial completeness increases progressively with each additional survey stage.

Successful integration of TLS and UAV datasets required careful planning and post-processing. Drone missions required appropriate overlap and lighting conditions, while dataset integration relied on noise filtering, subsampling, and precise ICP alignment. These steps ensured that the final HBIM dataset maintained high geometric accuracy despite variations in point density and viewpoint geometry^39^.

The study highlights the benefits of iterative workflows for heritage documentation. Rather than relying on a single comprehensive campaign, beginning with an initial survey, assessing quality, and selectively adding data where gaps remain proves more resource efficient and consistent with principles of minimal intervention in conservation projects. TLS remains essential for ground-level accuracy, while UAV photogrammetry effectively complements it by capturing elevated and occluded architectural features.

The findings are based on a single case study with specific access restrictions, dense vegetation, and seasonal conditions, and the evaluation used two TLS instruments (Leica HDS-6200 and BLK360 G2). The improvements in coverage and deviation metrics therefore reflect these site-specific circumstances. Although the PSFEM framework and its parameters, such as effective range, clearance angle, and environmental modifiers, provide a transferable structure, they should be recalibrated for different building typologies or urban environments where occlusion patterns vary. Future work should test the model across multiple sites, with attention to seasonal foliage and automated parameter selection, to enhance its generalizability.

## Proposed decision-making framework

Based on the outcomes of this study, a structured, phased decision-making framework to guide heritage professionals through an efficient hybrid HBIM reality capture workflow. This approach prioritizes data quality, resource efficiency, and adaptability, emphasizing iterative evaluation and targeted resurveying only, when necessary, rather than exhaustive one-time campaigns.

**Fig. 8:**
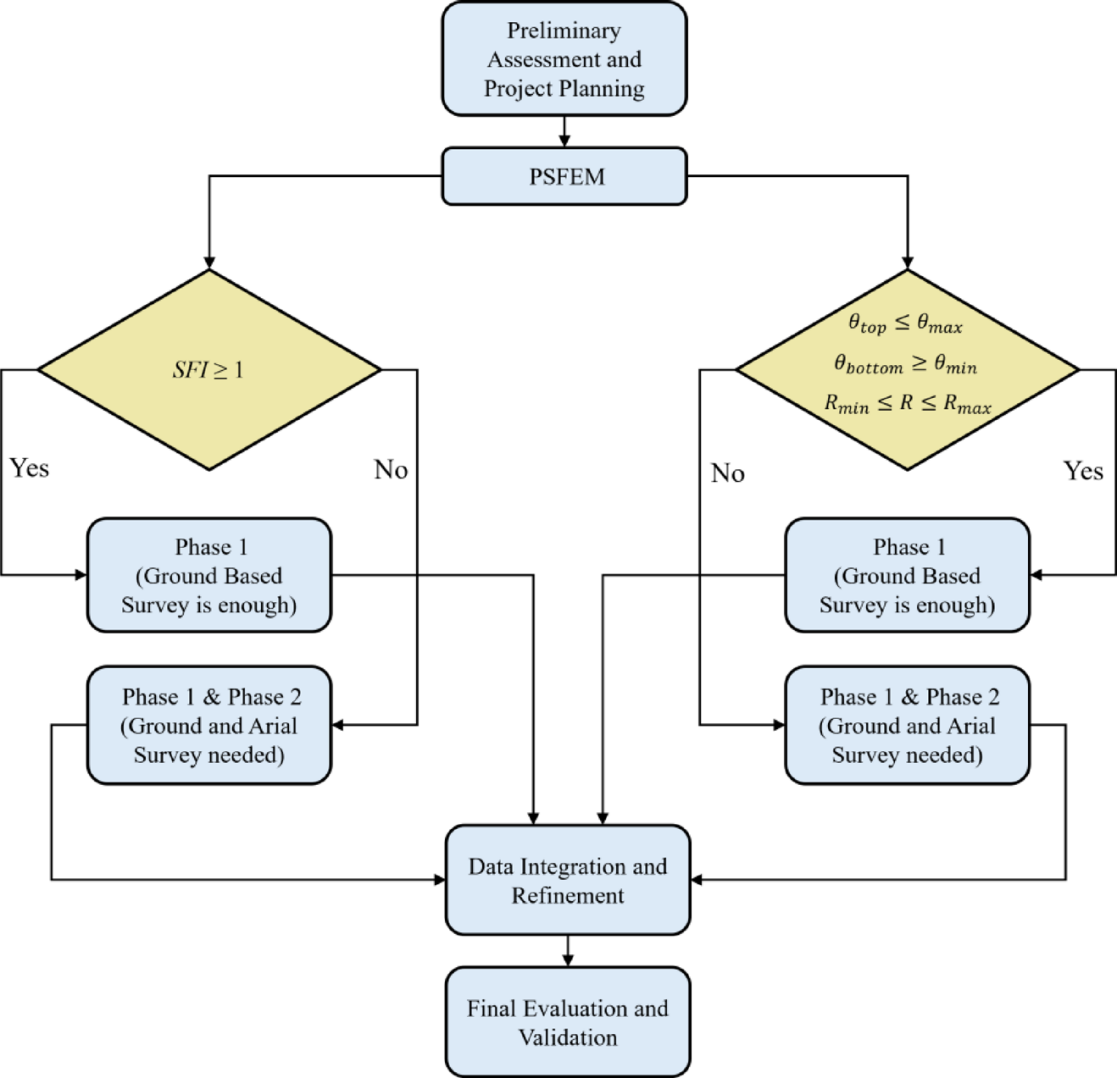
The decision-making workflow structure.

The framework consists of the following steps (Fig. 8):

1. Step 1: Preliminary Assessment and Project Planning
  Analyze site conditions, building geometry, and access constraints using available site documentation, photographs, or initial site visits.
  Define documentation goals (e.g., LOD requirements, conservation analysis, deformation monitoring) to align with project objectives.
  Plan required tools
2. Step 2: PSFEM

Apply the PSFEM to quantitatively confirm limitations related to ground-based capture, using parameters reflecting site-specific constraints. If surface coverage falls below acceptable thresholds or occluding areas exceed acceptable limits, and feasibility modeling confirms limitations, plan for a Phase 2 survey.

3. Step 3: (Phase 1) Initial Ground-Based Survey

Begin with TLS combined with ground-based photogrammetry to capture detailed geometry and rich visual textures in accessible areas. TLS provides high metric accuracy, while photogrammetry supplements texture detail and occlusion zones where camera access is feasible.

4. Step 4: (Phase 2) Aerial and Supplemental TLS Survey
  Conduct a second survey focused on identifying deficiencies:
  Deploy drone-based photogrammetry for high-angle and sloped surfaces.
  Add supplemental TLS scans in shadowed or occluded areas, such as behind projections or within narrow courtyards.
  This approach conserves resources while ensuring comprehensive coverage where it is most needed.
5. Step 5: Data Integration and Refinement
  Integrate and refine datasets to create a unified hybrid model:
  Register point clouds using ICP alignment for geometric consistency.
  Apply statistical outlier removal and smoothing filters to enhance surface quality.
  Perform visual inspections and localized adjustments to validate alignment and correct minor misalignments.
6. Step 6: Final Evaluation and Validation
  Use updated C2C deviation maps to confirm accuracy improvements.
  Ensure surface coverage exceeds 95%, particularly in areas identified as previously incomplete.
  Evaluate surface consistency and detail quality across the combined dataset.

This decision-making framework prioritizes advanced planning, structured phase determination, and adaptive deployment of tools, ensuring that HBIM documentation achieves high geometric accuracy and spatial completeness while minimizing time and resource expenditure. By guiding decision-making through clear, quantifiable criteria, the framework supports professionals in aligning survey phases with project timelines, particularly for heritage sites with access restrictions or conservation constraints.

This structured approach enables scalable and repeatable implementation in diverse heritage contexts, facilitating efficient, high-quality HBIM practices that align with conservation and documentation standards while ensuring effective timeline and resource management throughout project execution.

## Conclusion

This work confirms that a phased hybrid data acquisition approach, supported by predictive modeling, significantly enhances HBIM documentation, especially in complex heritage sites with restricted access.

By combining TLS, ground-based, and drone-based photogrammetry, this workflow overcomes the limitations of single-phase ground-only approaches. It enables comprehensive spatial coverage while preserving high geometric accuracy across critical architectural components^49–51^.

Although the PSFEM was developed after Phase 1 rather than used in advance, its basis in site-specific constraints demonstrates how future documentation campaigns can strategically determine the need for drone or supplementary TLS data, shifting from the traditional reactive approach to a “Predict Then Capture” paradigm.

Key quantitative outcomes from the Sopronhorpács Chapel case study include:

- Surface coverage increased from approximately 54% during the initial ground-based survey to 96% following targeted aerial and supplemental TLS data acquisition.
- Elevated and occluded architectural features were successfully documented while preserving accuracy.
- Iterative assessment using cloud-to-cloud deviation, occlusion mapping, and surface roughness analysis ensured that resurveying was conducted only when necessary, reducing redundant fieldwork while achieving comprehensive documentation.

This framework offers a practical and adaptable guide for heritage professionals implementing HBIM workflows at sites with access restrictions, conservation sensitivities, or complex geometries. It enables evidence-based, adaptive decision-making throughout the documentation process, ensuring that final HBIM models meet high standards of completeness and reliability while aligning with principles of minimal intervention in heritage conservation.

Looking ahead, future work will focus on validating the predictive scan feasibility estimation model across additional case studies to confirm its accuracy and usability in diverse heritage contexts. Further integration of AI-driven alignment, machine learning-based occlusion detection, and automated drone mission planning will enhance adaptability and efficiency. These advancements will support predictive, automated, and intelligent HBIM documentation workflows, producing robust digital records for the long-term conservation, monitoring, and analysis of cultural heritage structures, ensuring their preservation for future generations.

## Data Availability

The datasets used and/or analysed during the current study are available from the corresponding author on reasonable request.
